# Prevalence and Prognostic Impact of Deranged Liver Blood Tests in COVID-19: Experience from the Regional COVID-19 Center over the Cohort of 3812 Hospitalized Patients

**DOI:** 10.3390/jcm10184222

**Published:** 2021-09-17

**Authors:** Frane Paštrovic, Marko Lucijanic, Armin Atic, Josip Stojic, Mislav Barisic Jaman, Ida Tjesic Drinkovic, Marko Zelenika, Marko Milosevic, Barbara Medic, Jelena Loncar, Maja Mijic, Tajana Filipec Kanizaj, Dominik Kralj, Ivan Lerotic, Lucija Virovic Jukic, Neven Ljubicic, Kresimir Luetic, Dora Grgic, Matea Majerovic, Rajko Ostojic, Zeljko Krznaric, Ivica Luksic, Nevenka Piskac Zivkovic, Tatjana Keres, Vlatko Grabovac, Jasminka Persec, Bruno Barsic, Ivica Grgurevic

**Affiliations:** 1 Department of Gastroenterology, Hepatology and Clinical Nutrition, University Hospital Dubrava, 10000 Zagreb, Croatia; fpastrovic@gmail.com (F.P.); mislav.barisic.jaman@gmail.com (M.B.J.); ida.tjesicdrinkovic@gmail.com (I.T.D.); markozelenika@gmail.com (M.Z.); markom342@gmail.com (M.M.); barby.medic@gmail.com (B.M.); jloncarzg@gmail.com (J.L.); 2 Department of Hematology, University Hospital Dubrava, 10000 Zagreb, Croatia; markolucijanic@yahoo.com; 3 Department of Gastroenterology, Hepatology and Clinical Nutrition, School of Medicine, University of Zagreb, 10000 Zagreb, Croatia; tajana.filipec@gmail.com (T.F.K.); lucija.jukic@gmail.com (L.V.J.); neven.ljubicic@kbcsm.hr (N.L.); rajko.ostojic@gmail.com (R.O.); zeljko.krznaric1@zg.ht.hr (Z.K.); luksic.ivica@gmail.com (I.L.); barsicbruno@gmail.com (B.B.); 4 Department of Emergency Medicine, University Hospital Dubrava, 10000 Zagreb, Croatia; aticarmin@gmail.com (A.A.); josip.stojic95@gmail.com (J.S.); gr.vlatko@gmail.com (V.G.); 5 Department of Gastroenterology and Hepatology, University Hospital Merkur, 10000 Zagreb, Croatia; mijic.maja@gmail.com; 6 Department of Gastroenterology and Hepatology, University Hospital Sestre Milosrdnice, 10000 Zagreb, Croatia; dominik.rex@gmail.com (D.K.); ivanlerotic@yahoo.com (I.L.); 7 Department of Gastroenterology and Hepatology, University Hospital Sveti Duh, 10000 Zagreb, Croatia; kresimir.luetic@hlk.hr; 8 Department of Gastroenterology and Hepatology, University Hospital Center Zagreb, 10000 Zagreb, Croatia; dora.grgic1@gmail.com (D.G.); matea.majerovic@gmail.com (M.M.); 9 Department of Maxillofacial Surgery, University Hospital Dubrava, 10000 Zagreb, Croatia; 10 Department of Pulmology, University Hospital Dubrava, 10000 Zagreb, Croatia; npiskac@gmail.com; 11 Intensive Care Unit, Department of Internal Medicine, University Hospital Dubrava, 10000 Zagreb, Croatia; tatjana.keres@gmail.com; 12 Intensive Care Unit, Department of Anestesiology, Renimatology and Intensive Care, University Hospital Dubrava, 10000 Zagreb, Croatia; anestezija.predstojnica@kbd.hr; 13 Department of Anestesiology, Renimatology and Intensive Care, School of Dental Medicine, University of Zagreb, 10000 Zagreb, Croatia; 14 Faculty of Pharmacy and Biochemistry, University of Zagreb, 10000 Zagreb, Croatia

**Keywords:** COVID-19, liver functional tests, mortality

## Abstract

Background: Derangement of liver blood tests (LBT) is frequent in patients with Coronavirus disease 2019 (COVID-19). We aimed to evaluate (a) the prevalence of deranged LBT as well as their association with (b) clinical severity at admission and (c) 30-day outcomes among the hospitalized patients with COVID-19. Methods: Consecutive patients with COVID-19 hospitalized in the regional referral center over the 12-month period were included. Clinical severity of COVID-19 at hospital admission and 30-day outcomes (need for intensive care, mechanical ventilation, or death) were analyzed. Results: Derangement of LBT occurred in 2854/3812 (74.9%) of patients, most frequently due to elevation of AST (61.6%), GGT (46.1%) and ALT (33.4%). Elevated AST, ALT, GGT and low albumin were associated with more severe disease at admission. However, in multivariate Cox regression analysis, when adjusted for age, sex, obesity and presence of chronic liver disease, only AST remained associated with the risk of dying (HR 1.5081 and 2.1315, for elevations 1–3 × ULN and >3 × ULN, respectively) independently of comorbidity burden and COVID-19 severity at admission. Patients with more severe liver injury more frequently experienced defined adverse outcomes. Conclusions: Deranged LBTs are common among patients hospitalized with COVID-19 and might be used as predictors of adverse clinical outcomes.

## 1. Introduction

Coronavirus disease 2019 (COVID-19) is a multisystemic disease, with pandemic features [1]. The clinical picture is characterized by respiratory symptoms of various severity, including the development of pneumonia and respiratory failure with the need for oxygen supplementation, and these patients require hospital admission. Further deterioration in the form of acute respiratory distress syndrome (ARDS) and need for mechanical ventilation (MV) occurs in around 15–20% of hospitalized patients [1,2,3]. Along with respiratory illness other organs and systems are affected, including coagulation with the development of thromboembolic incidents, bleeding, myocarditis, central and peripheral nervous system affection, musculoskeletal symptoms, gastrointestinal and hepatobiliary problems [3].

Whereas elevation of aminotransferases has been commonly seen in hospitalized patients with COVID-19, liver failure represents a rare development usually encountered among patients with already known liver cirrhosis, or as the part of multiorgan failure caused by severe inflammatory response syndrome and septic shock [4,5,6,7,8,9]. In some previous reports deranged liver blood tests (LBT) were associated with more severe forms of COVID-19 and adverse clinical outcomes, although not all the authors came to the same conclusion [10,11,12,13,14]. As elevated aminotransferases are not organ specific, they might originate not only from the liver but also from muscles and other sources, and in line with rare occurrence of liver failure, some authors argue about the clinical importance of elevated aminotransferases and about the potential liver involvement in COVID-19 [15,16]. This view is supported by the lack of larger series of liver biopsies, and the microinjury of muscles in COVID-19 leading to the elevation of aminotransferases [17]. Additionally, studies that were using liver dedicated non-invasive diagnostic devices such of Fibroscan came to conflicting conclusions in terms of liver involvement and prognostic impact of the indicators of liver health among patients with COVID-19 [18,19]. 

Therefore, we aimed to evaluate (a) prevalence of deranged LBT at admission to hospital, as well as their association with (b) clinical severity, and (c) 30-day outcomes among the hospitalized patients with COVID-19, reflecting the real-life experience from the largest regional COVID-19 hospital in Croatia. 

## 2. Patients and Methods

### 2.1. Patients

This study included 3.812 consecutive patients with COVID-19 who were hospitalized in Dubrava University Hospital over the period from 19 March 2020 to 19 March 2021. Dubrava University Hospital was completely re-purposed to serve exclusively as the regional tertiary COVID-19 center during COVID-19 pandemic. 

All patients had a positive nasopharyngeal swab on severe acute respiratory syndrome coronavirus 2 (SARS-CoV-2) by polymerase chain reaction (PCR) or antigen test and were admitted through the hospital’s emergency department. Standardized clinical work-up was performed for each patient, including medical history taking, clinical examination, obtaining blood biochemistry, peripheral oxygen saturation, chest X-ray, and electrocardiogram. Other examinations were performed as indicated based on the clinical picture and decision of the attending physician. Included were the patients with available laboratory and clinical parameters collected within the 24 h from admission, sufficient to assess the severity of COVID-19, presence of comorbidity, along with LBTs (aspartate aminotransferase (AST), alanine aminotransferase (ALT), gamma-glutamyl transferase (GGT), alkaline phosphatase (ALP), total bilirubin (Bil), serum albumin (alb), prothrombin time (PT)), complete blood count (CBC), who were followed for 30-day from the admission to hospital. 

Patients were admitted to the intensive care unit (ICU), or to the regular ward based on the severity of clinical picture at presentation, as assessed in emergency department according to the national guidelines that incorporated severity of pneumonia and modified early warning score (MEWS) [20,21]. Upon admission to the ward, patients were treated with corticosteroids, antivirals (hydroxychloroquine, ritonavir/lopinavir or remdesivir), low molecular weight heparin (LMWH) and oxygen supplementation as needed according to the national guidelines. LMWH was prescribed in prophylactic doses to all patients without contraindication, whereas therapeutic doses were used in patients with documented thromboembolic events, as well as in those with elevated D-dimers upon judgement of the attending physician, especially in more severe forms of COVID-19. Other medications (for chronic medical conditions and acute complications, including antimicrobials) were administrated upon the decision of the attending physician on the ward. Antibiotics were not a part of the standardized initial treatment of COVID-19. Oxygen was delivered by bi-nasal catheters, masks (up to 15 L/min) or high-flow nasal cannula (HFNC, if >15 L/min was needed). All presented patients completed their hospitalization for acute COVID-19. This paper is a part of the project “Registar hospitalno liječenih bolesnika u Respiracijskom centru KB Dubrava”/“Registry of patients hospitalized in University Hospital Dubrava Respiratory center”.

### 2.2. Methods

Severity of COVID-19 at admission was graded using the classification from the national guidelines for treatment of COVID-19, version 2, issued on 19 November 2020 by the Ministry of Health [20]. Severe COVID-19 was considered in patients presenting with (a) bilateral pneumonia accompanied by either of the following features: (i) respiration rate (RR) ≥ 30/min; (ii) respiratory failure, (iii) peripheral oxygen saturation ≤ 93% (in resting state, room air); or (b) MEWS 3–4. Critical form of disease was considered in patients with (a) ARDS (partial pressure of arterial oxygen/fraction of inspired oxygen (PaO_2_)/FiO_2_) ≤ 300 mmHg), (b) presence of sepsis or septic shock, with/without organs’ failure, or (c) MEWS ≥ 5, and these patients required ICU admission. Comorbidities were assessed as individual entities and were summarized using the Charlson comorbidity index [22]. Eastern Cooperative Oncology Group (ECOG) score was used to assess the overall physical performance [23]. Obesity was defined as body mass index (BMI) > 30 kg/m^2^. All LBTs were recorded at hospital admission. The following were considered as normal ranges: Bilirubin ≤ 20 µmol/L, AST ≤ 30 IU/L in females and ≤ 38 IU/L for males, ALT ≤ 36 for females and ≤48 for males, GGT ≤ 35 IU/L for females and ≤ 55 IU/L for males, ALP ≤ 153 IU/L for females and ≤ 142 IU/L for males, albumin ≥ 35 g/L, and PT (quick) ≥ 70%. In accordance to the proposed nomenclature, the term LBT referred to all these tests, whereas the term “liver enzymes” referred to AST, ALT, GGT and AP [24]. “Liver injury” was considered in patients having deranged any of the liver enzymes accompanied by the elevated bilirubin. We did not use albumin or PT to define the presence of liver injury, as the duration of the disease at presentation was too short to result in decreased level of albumin, and many of patients had low albumin level and PT due to co-morbidity or nutritional issues. In addition, many patients used oral anticoagulants. Venous (pulmonary embolism, deep vein thrombosis) and arterial (myocardial infarction, cerebrovascular insult, peripheral embolization, mesenterial thrombosis) thrombotic events were considered only if documented by objective imaging and laboratory methods. Bleeding (gastrointestinal, epistaxis/hemoptysis, intramuscular, hematuria, retroperitoneal, intracranial) was considered as clinically relevant if documented in medical documentation. Thirty-day mortality was assessed from the date of hospital admission.

### 2.3. Statistical Methods

The presented analyses are retrospective in nature. Normality of distribution of numerical variables was tested using the Kolmogorov–Smirnov test. All numerical variables were non-normally distributed and were presented as median and interquartile range (IQR) and were compared between groups using the Mann–Whitney U or the Kruskal–Wallis ANOVA test where appropriate. The Jonckheere–Terpstra test for trend was used to assess rising or degrading trends of specific parameters over disease severity categories. Correlation between numerical variables was assessed using the Spearman rank correlation, relationships with Rho ≥ 0.2 considered as meaningful. Categorical variables are presented as frequency and percentage and were compared between groups using the Χ^2^ test and the Χ^2^ test for trend. Survival analyses were based on the Kaplan–Meier method. Survival curves were compared using the Cox–Mantel version of the log-rank test for univariate and the Cox regression analysis for multivariate analyses. The log-rank test for trend was used for assessing the trend of gradual increase in mortality with higher degree of derangement of liver specific parameters. *p*-values < 0.05 were considered statistically significant. All analyses were performed using the MedCalc statistical software version 20 (MedCalc Software Ltd., Ostend, Belgium).

### 2.4. Ethical Issues

This study was conducted in accordance with the World Medical Association Declaration of Helsinki and the study protocol was approved by the Institutional Ethics Committee, No. 2020/1012-10. Due to retrospective design signed informed consent was waived by the Ethics Committee.

## 3. Results

### 3.1. Patients’ Characteristics

A total of 3812 COVID-19 patients were analyzed. Median age was 74 years, IQR (64–82). There were 2148/3812 (56.3%) males and 1664/3812 (43.7%) females, and 1023 (28.6%) of patients were obese. There was a significant burden of co-morbidities as 2658 (69.7%), 1154 (30.3%), 617 (16.2%) and 474 (12.4%) patients had arterial hypertension, diabetes, congestive heart failure and chronic kidney disease, respectively. One hundred and six (2.8%) of the patients had history of chronic liver disease, and of them 49 (1.3%) had cirrhosis. Four hundred and thirty-four (11.4%) patients were current smokers. A total of 3390 (88.9%) patients had pneumonia at admission, and of them 3136 (82.3%) required oxygen supplementation. Median length of hospitalization was 10 days IQR (6–16). Rates of ICU admission, need for HFNC oxygenation and MV were 23.1%, 19.9% and 17.3%, respectively. A total of 1315 (34.5%) patients died during the 30-day period. Patients’ characteristics at admission to hospital and their outcomes are shown in Table 1. The relationship between the patients’ characteristics with LBT profile is shown in Supplementary Table S1.

### 3.2. Relationship between Patients’ Characteristics and the Profile of LBTs at Admission

Median AST levels were 41 IQR (28–64). A total of 1432 (38.4%), 1922 (51.5%) and 377 (10.1%) patients presented with normal, 1–3 × elevated and >3 × elevated AST levels on admission, respectively. Higher AST was significantly associated with male sex, obesity, presence of chronic liver disease and liver cirrhosis, higher CRP, and higher ferritin (*p* < 0.05 for all analyses). 

Median ALT levels were 31 IQR (19–52). A total of 2521 (67.4%), 1049 (28%) and 172 (4.6%) patients presented with normal, 1–3 × elevated and >3 × elevated ALT levels on admission, respectively. Higher ALT was significantly associated with male sex, younger age, obesity, with active or previous smoking, higher hemoglobin, and higher ferritin (*p* < 0.05 for all analyses). 

Median GGT levels were 42 IQR (24–81). A total of 1934 (53.9%), 1212 (33.8%) and 441 (12.3%) patients presented with normal, 1–3 × elevated and >3 × elevated GGT levels on admission, respectively. Higher GGT was significantly associated with younger age, male sex, obesity, presence of chronic liver disease and liver cirrhosis, alcohol use, active or previous smoking, use of pre-admission antibiotic therapy, and with higher ferritin (*p* < 0.05 for all analyses). 

Median ALP levels were 72 IQR (56–97). A total of 2862 (90.3%), 270 (8.5%) and 39 (1.2%) patients presented with normal, 1–3 × elevated and >3 × elevated ALP levels on admission, respectively. Higher ALP was significantly associated with non-obesity, presence of chronic kidney disease, presence of chronic liver disease and liver cirrhosis, use of oral anticoagulant therapy, use of pre-admission antibiotic therapy, and with higher D-dimers (*p* < 0.05 for all analyses). 

Median total bilirubin levels were 11.4 IQR (8.6–15.9). A total of 2586 (85.3%), 375 (12.4%) and 69 (2.3%) patients presented with normal, 1–3 × elevated and >3 × elevated total bilirubin levels on admission, respectively. Higher total bilirubin was significantly associated with male sex, presence of congestive heart failure, presence of chronic liver disease and liver cirrhosis, alcohol use, oral anticoagulant therapy use (*p* < 0.05 for all analyses). 

Median albumin levels were 32 g/L IQR (28–35). A total of 114 (4.9%), 515 (22.1%) and 1698 (73%) patients presented with albumin levels of ≥40 g/L, 35–39 g/L and <35 g/L, respectively. Lower albumin was significantly associated with older age, female sex, arterial hypertension, congestive heart failure, chronic kidney disease, chronic liver disease and liver cirrhosis, higher Charlson comorbidity index, active or previous smoking, use of oral anticoagulant therapy, use of pre-admission antibiotic therapy, higher WBC, lower hemoglobin, higher CRP, higher ferritin, and higher D-dimers (*p* < 0.05 for all analyses). 

Median PT values were 100% IQR (89–109%). Considering that 27.5% of patients were receiving oral anticoagulant therapy, further analysis was narrowed to the subgroup without exposure to these drugs. Accordingly, lower PT values were significantly associated with male sex, presence of congestive heart failure, presence of chronic liver disease and liver cirrhosis, use of pre-admission antibiotic therapy, lower hemoglobin, and higher D-dimers (*p* < 0.05 for all analyses). 

Associations of each of the analyzed LBTs with other parameters were either non-significant or associated with very low coefficient of correlation (<0.2) to be considered meaningful. 

### 3.3. Relationship of LBTs with COVID-19 Severity at Admission

Median time from the first symptoms of COVID-19 to admission was 5 days IQR (1–9). Only higher ALT was associated with longer disease duration prior to admission, whereas lower albumin was associated with worse ECOG functional status. Higher AST, ALT, GGT and lower ALP, albumin and PT were associated with severe clinical presentation of COVID-19 (*p* < 0.05 for all analyses). However, total bilirubin showed no association with the COVID-19 severity at admission. 

### 3.4. Associations between Deranged LBTs at Admission and Clinical Outcomes

Associations of clinical outcomes with LBTs are shown in Table 2. Higher AST, ALT, GGT and lower albumin were significantly associated with ICU admission, need for HFNC oxygenation and MV, higher total bilirubin was associated with the ICU admission only (*p* < 0.05 for all analyses), whereas ALP and PT show no associations with these outcomes (*p* > 0.05). When analyzed as continuous variables, higher AST, ALP, bilirubin and lower albumin and PT were associated with inferior 30-day survival, while ALT and GGT were not associated with inferior 30-day survival (*p* < 0.05 for all analyses).

**Figure 1:**
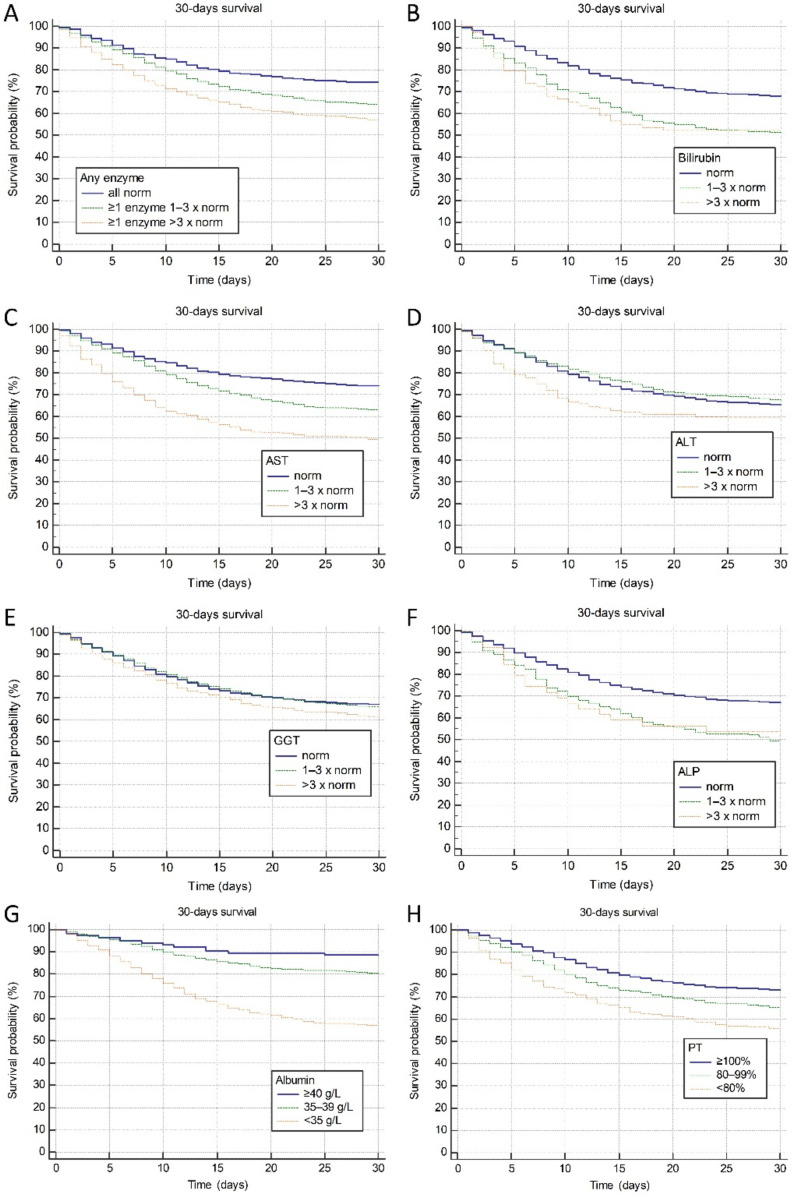
Association of the degree of liver blood tests derangement and 30-day mortality. (**A**) elevation in any of AST, ALT, GGT, ALP, total bilirubin. Elevation in (**B**) total bilirubin, (**C**) AST, (**D**) ALT, (**E**) GGT, (**F**) ALP, (**G**) reduction in albumin and (**H**) PT. Legend: AST—aspartate aminotransferase, ALT—alanine aminotransferase, GGT—gamma-glutamyl transferase, ALP—alkaline phosphatase, PT—prothrombin time (quick, %).

We further investigated associations of LBTs with 30-day mortality using the time to event survival analyses stratified by the degree of derangement from normal values (normal, 1–3 × elevated and >3 × elevated for AST, ALT, GGT, ALP and bilirubin; ≥40 g/L, 35–39 g/L and <35 g/L for albumin, and ≥100%, 80–99% and <80% for PT). As depicted in Figure 1A–F, significant gradual increase in mortality was observed with higher degree of derangement of all investigated parameters except for ALT (*p* for trend <0.05 for all analyses except for ALT). Hazard ratios and confidence intervals for the associations of each LBT with 30-day mortality stratified categorically by the degree of derangement are presented in Table 3. 

Considering the relationship of LBTs with 30-day mortality, a Cox regression analysis model controlling for age, sex, obesity, Charlson comorbidity index, MEWS severity, chronic liver disease, liver cirrhosis, AST, ALT, GGT, ALP, total bilirubin, albumin and PT was created to assess independent associations. Model is shown in Table 4. As presented, AST elevation 1–3 × ULN and >3 × ULN was negatively and ALT elevation 1–3 × ULN was positively associated with 30-day survival independently of each other and age, comorbidity burden and disease severity at presentation.

### 3.5. Associations between the Presence of Liver Injury at Admission and Clinical Outcomes

**Figure 2:**
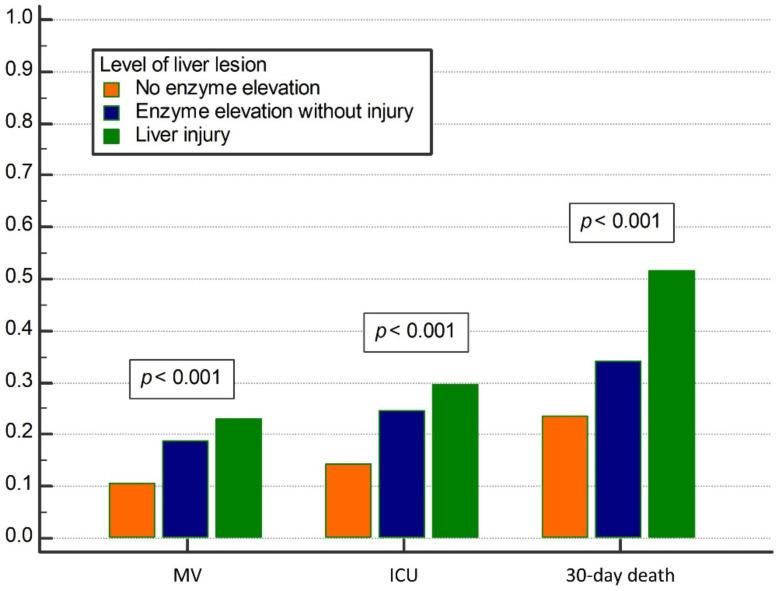
Relationship between the level of liver lesion and clinical outcomes (mechanical ventilation (MV), Intensive care unit (ICU) admission, and 30-day mortality). Liver injury was defined as the elevation of any liver enzyme plus elevated bilirubin. Liver enzymes: aspartate aminotransferase, alanine aminotransferase, gamma-glutamyl transferase, alkaline phosphatase.

A total of 2650 patients had available synchronous data on all liver enzymes (AST, ALT, GGT, ALP) and bilirubin among whom a total of 314 (11.8%) had combined elevation of both bilirubin and any of the enzymes corresponding to the liver injury, 1732 (65.4%) had enzyme elevation without elevated bilirubin, and in 604 (22.8%) neither enzymes nor bilirubin were elevated. There was a significant trend of increased frequency of adverse outcomes (MV, ICU admission, 30-day mortality) over higher degree of liver lesion (*p* < 0.001 for overall difference and *p* < 0.001 for trend for all outcomes, Figure 2). Patients with normal levels of liver enzymes and bilirubin, those with elevated enzymes but not bilirubin, and patients with liver injury needed MV in 10.6%, 18.9% and 22.9% cases, respectively, were transferred to ICU in 14.4%, 24.5% and 29.6% cases, respectively and experienced death during 30-days of hospitalization in 23.7%, 34.2% and 51.6% cases, respectively.

## 4. Discussion

This study analyzed derangements of LBTs in one of the largest cohort of patients hospitalized with COVID-19 reported so far, as well as their association with the clinical severity at admission and 30-day outcomes. Derangement of LBTs occurred in 3/4 of patients at admission to hospital, most frequently due to the elevation of AST, followed by GGT and ALT. Elevated AST, ALT, GGT and low ALP, albumin and PT were associated with more severe disease at admission, whereas only elevated AST was independent predictor of death. There is incremental trend for higher rates of ICU admission, MV and 30-day mortality among the patients with liver injury when compared to those with only deranged liver enzymes and those with normal liver biochemistry. 

According to the presented results elevated LBTs are frequently observed among patients with COVID-19 at admission to hospital. Elevation of at least one LBT was observed in almost 75% of patients in our cohort at admission. In line with other reports AST was the most frequently elevated (in 61.6%), followed by GGT in 46.1% and ALT in 33.4% of patients. This is much higher prevalence of deranged LBTs than initially reported in studies coming from China (14.9%), but comparable to data from United States ranging from 40–67% [1,2,3,25,26]. The observed differences in the rates of elevated LBTs might be due to some racial specificities, different threshold for hospital admission and the prevalence of severe cases, but also due to demographic features including age, prevalence of obesity, drinking habits, chronic medication use, just to mention the most obvious reasons. Indeed, three most frequently elevated LBTs in our population (AST, GGT, ALT) share the common denominators as they were all associated with male sex, presence of obesity and elevated ferritin level. In addition, elevated GGT was associated with alcohol use, and both ALT and GGT with smoking. These associations might point to the underreported alcohol consumption and unrecognized prevalence of non-alcoholic fatty liver disease in the analyzed cohort, reflecting their prevalence in general population. On the other hand, association with the inflammatory mediators (ferritin, and in case of AST with CRP as well), may link these enzymes with the liver involvement in the inflammatory response to COVID-19. The source of elevated LBTs in COVID-19 has been repeatedly discussed, as they are not completely liver-specific and may originate from other tissues, such as muscles [15,16]. However, other authors did not find consistent correlation between elevated AST and markers of muscle injury, leading to conclusion that the liver was the most likely source of elevated LBTs [4]. This liver lesion seems not to be clinically significant in majority of cases, as liver failure develops only exceptionally, usually among the patients with already compromised liver function due to existing cirrhosis, or as the part of multiorgan failure in most severe cases of COVID-19 [6,7,17].

Deranged LBTs are associated with more severe clinical presentation of COVID-19, which is in keeping with the reports from other authors [5,10,25]. In our cohort, more severe clinical presentation of COVID-19 at admission was observed among patients with elevated AST, ALT, GGT and low ALP, albumin and PT. Whereas elevated liver enzymes suggest the presence of strong inflammatory response with liver involvement, decreased PT and albumin most probably reflect the presence of comorbidity, worse nutritional and overall performance status, and therefore these associations appear logical. However, a word of caution is needed, as the design of this study does not allow for definitive conclusion about the association between deranged LBTs and severity of COVID-19. Namely, LBTs were analyzed only at the admission to hospital and not at the peak hospitalization/peak of illness. In some previous studies, further increase in frequency and the level of LBTs was reported in patients who developed more severe clinical picture, even if they had mild disease at admission. Additionally, patients presented with variable durations of symptoms before admission (IQR 1–9 days), which represents additional bias.

The most important result of our study is clear association between the serum levels of LBTs and clinical outcomes, indicating strongly for their prognostic significance. Indeed, significant gradual increase in mortality was observed with higher degree of derangement of all investigated LBTs, except for ALT (Figure 1). However, the risk of dying from COVID-19 was independently increased only among the patients with elevated AST at admission (depending on the magnitude of AST elevation), and this association persisted after adjustment for age, sex, obesity, comorbidity, disease severity at admission, and other investigated LBTs. Interestingly, mild (<3 × ULN) elevation of ALT was found protective in terms of mortality, whereas ALT > 3 × ULN re-gained detrimental effect on survival. We do not see a mechanistic explanation for this protective effect of mildly elevated ALT, and the same phenomenon was reported in a recently published study from China [27].

Conflicting data have been published regarding the prognostic impact of elevated LBTs. Whereas in some studies, elevated LBTs were associated with more severe clinical presentation but they were lacking follow-up data to analyze mortality, and others reported various associations to ICU admission, need for mechanical ventilation and death [5,7,10,11,12,13,14,25]. In the study conducted over the 1827 hospitalized patients in United States, higher risk of dying was observed only among patients with elevated baseline bilirubin, whereas both elevated bilirubin and AST as recorded at the peak hospitalization were associated with death, and other LBTs were not [25]. However, abnormal AST, Bil and albumin at admission were all associated with the higher risk of ICU admission and mechanical ventilation. 

The pathophysiological role of the liver in COVID-19 remains still not fully elucidated, as histological data are scarce, due to very infrequent liver biopsy taking. Whereas ACE2 receptors, as the gate for SARS-CoV-2 entry in the cell, are most abundantly expressed on the cholangiocytes, hepatocellular profile of elevated LBTs has been commonly reported, and histological changes (in the limited series of patients) were more in favor of deranged liver circulation (microthrombosis), mitochondrial dysfunction and mild hepatitis, rather than biliary injury [4,6,28,29]. In keeping with these findings, it is not unexpected that AST is most frequently elevated in patients with COVID-19, as it has been considered specific for liver ischemia, mitochondrial dysfunction and alcohol related liver disease [4,6]. Yet, reliable evidence of viral replication within hepatocytes has not been confirmed, so there is still doubt if liver lesion is caused by virus itself or is it immune-mediated [6,30,31]. As for the biliary injury, cases of severe post-COVID cholangiopathy with some distinct pathological features have recently been reported among patients who suffered from critical disease and were mechanically ventilated [32].

Liver involvement in pathogenesis of COVID-19 and prognostic importance of LBTs is furtherly supported by our results showing incremental increase in frequency of adverse outcomes (ICU admission, MV or death) when patients were assessed according to the severity of liver lesion. Indeed, patients with liver injury (defined here by elevated bilirubin in addition to elevated liver enzymes) had worse prognosis when compared to patients with the isolated elevation of liver enzymes (not accompanied by the elevated bilirubin), and to patients with normal both liver enzymes and bilirubin. Based on our results and available data from other studies we believe it is still not possible to claim if liver contributes to the severity of inflammatory response to SARS CoV-2 infection, and hence to the severity of COVID-19, or is it only indicator and the part of generalized severe inflammatory response to the virus. In any case, patients with biochemical indicators of more severe liver injury appear to be under increased risk of severe clinical course including the need for ICU treatment, MV and death.

In contrast to the high prevalence of biochemical abnormalities suggestive of liver involvement in COVID-19, the prevalence of chronic liver disease (2.8%) and cirrhosis (1.3%) observed in our cohort was low [3,19]. We assume this might be due to the awareness of the risks of acquiring COVID-19 among liver patients and preventive measures taken to avoid exposure to infection. 

Interestingly, the association between the presence of cirrhosis and mortality risk was not independent when adjusted to other covariables in multivariate regression analysis as shown in Table 4. We assume that cirrhosis could not achieved statistical significance in the context of deranged LBT, probably due to overlapping prognostic properties and insufficient statistical power as the result of low number of patients with cirrhosis (*n* = 49), as compared to the entire cohort of 3812 patients analyzed here.

High mortality in our cohort might result from the selection criteria for the admission, as our hospital was the major tertiary referral center taking care of patients with most severe forms of COVID-19, as well as for those with other urgent conditions complicated with COVID-19. Indeed, almost 90% of patients had pneumonia, 82% required oxygen supplementation, and at peak pandemic almost 25% needed ICU admission or were receiving HFNC oxygenation outside ICU. In addition, the analyzed population was old, with median age of 74 and burdened by multiple co-morbidities. 

The limitations of our study are single center experience, selection of most severe/critical COVID-19 cases, or COVID-19 cases with comorbidities that required hospital level of care, which are representative of the tertiary COVID-19 hospital, and inability to longitudinally assess dynamics of particular measurements over time. In addition to this, we did not regularly perform liver imaging in patients with deranged LBT to furtherly explore liver status, given almost universal prevalence and self-limited course of deranged liver biochemistry in typical cases. Nevertheless, this study was performed in the one of the largest cohort of patients reported so far, representative for the Caucasian population with almost universal prevalence of pneumonia, all of whom underwent standardized diagnostic procedures and therapy. Therefore, the obtained results might be considered robust, with high statistical power.

In conclusion, patients who are hospitalized due to COVID-19 usually have elevated LBTs at admission. Only elevated AST is the independent predictor of death. There is an incremental trend for higher rates of ICU admission, MV and 30-day mortality among the patients with more severe liver injury when compared to those with normal liver biochemistry. 

## Figures and Tables

**Table 1:** Characteristics of the analyzed cohort of patients with COVID-19 at admission to hospital.

	N (%), Median (IQR)
Total number of patients	3812
Age (years)	74 (64–82)
Sex	
Female	1664 (43.7%)
Male	2148 (56.3%)
Arterial hypertension	
Yes	2658 (69.7%)
No	1154 (30.3%)
Diabetes mellitus	
Yes	1154 (30.3%)
No	2658 (69.7%)
Obesity (Body mass index ≥ 30 kg/m^2^)	
Yes	1023 (28.6%)
No	2554 (71.4%)
Congestive heart failure	
Yes	617 (16.2%)
No	3195 (82.8%)
Chronic kidney disease	
Yes	474 (12.4%)
No	3338 (87.6%)
Chronic liver disease	
Yes	106 (2.8%)
No	3706 (97.2%)
Liver cirrhosis	
Yes	49 (1.3%)
No	3763 (98.7%)
Charlson comorbidity index	4 IQR (3–6)
Alcohol use	
Yes	207 (5.4%)
No	3605 (94.6%)
Smoking	
Yes	434 (11.4%)
No	3378 (88.6%)
Number of drugs in chronic therapy	5 (3–8)
Statin use	
Yes	911 (23.9%)
No	2901 (76.1%)
Antibiotic therapy before admission	
Yes	1285 (33.7%)
No	2527 (66.3%)
Oral anticoagulant therapy	
Yes	1049 (27.5%)
No	2763 (72.5%)
AST (U/L)	41 (28–64)
ALT (U/L)	31 (19–52)
GGT (U/L)	42 (24–81)
ALP (U/L)	72 (56–97)
Total bilirubin (umol/L)	11.4 (8.6–15.9)
Albumin (g/L)	32 (28–35)
Prothrombin time (%, Quick) *	100% (89–109%)
Liver blood tests (any)	
Normal level	958 (25.1%)
Deranged	2854 (74.9%)
White blood cell count (×10^9^/L)	8 (5.8–11.2)
Hemoglobin (g/L)	128 (113–141)
Platelets (×10^9^/L)	221 (163–297)
C-reactive protein (mg/L)	88.7 (39.5–151)
Ferritin (ug/L)	711 (386–1289)
D-dimers (mg/L)	1.42 (0.73–3.6)
Day of disease on admission	5 (1–9)
ECOG status	3 (1–4)
Pneumonia	
Yes	3390 (88.9%)
No	422 (11.1%)
Oxygen therapy	
Yes	3136 (82.3%)
No	676 (17.7%)
MEWS severity	
Mild	392 (10.3%)
Moderate	196 (5.1%)
Severe	2652 (69.6%)
Critical	572 (15%)
ICU admission	
Yes	881 (23.1%)
No	2931 (76.9%)
Mechanical ventilation	
Yes	659 (17.3%)
No	3153 (82.7%)
30-day mortality	
Yes	1315 (34.5%)
No	2497 (65.5%)

Table legend: AST—aspartate aminotransferase, ALT—alanine aminotransferase, GGT—gamma-glutamyl transferase, ALP—alkaline phosphatase, IQR—interquartile range, ICU—intensive care unit, MEWS—modified early warning score, ECOG—Eastern Cooperative Oncology Group. * Prothrombin time values calculated for the patients not receiving oral anticoagulants.

**Table 2:** Relationship of liver blood tests with the clinical outcomes of hospitalized patients with COVID-19.

	Overall/Any Enzyme	AST (U/L)	ALT (U/L)	GGT (U/L)	ALP (U/L)	Bilirubin (umol/L)	Albumin (g/L)	PT (%, Quick)
Length of hospitalization	10 IQR (6–16)	Rho = 0; *p* = 0.852	Rho = −0.01; *p* = 0.516	Rho = 0.03; *p* = 0.111	Rho = 0; *p* = 0.965	Rho = −0.02; *p* = 0.141	Rho = −0.06; *p* = 0.008 *	Rho = 0.01; *p* = 0.534
Intensive care unit		Median	Median	Median	Median	Median	Median	Median
Yes	879 (23.1%)	47.5	34	47	72	11.7	30	100%
No	2933 (76.9%)	39	29	40	72	11.3	32	101%
		*p* < 0.001 *	*p* < 0.001 *	*p* < 0.001 *	*p* = 0.486	*p* = 0.022 *	*p* < 0.001 *	*p* = 0.283
High-flow oxygenation		Median	Median	Median	Median	Median	Median	Median
Yes	758 (19.9%)	49	35	51	72	11.5	31	102%
No	3054 (80.1%)	39	29	40	72	11.3	32	101%
		*p* < 0.001 *	*p* < 0.001 *	*p* < 0.001 *	*p* = 0.829	*p* = 0.481	*p* < 0.001 *	*p* = 0.528
Mechanical ventilation		Median	Median	Median	Median	Median	Median	Median
Yes	660 (17.3%)	48	34	49	73	11.8	30	101%
No	3152 (82.7%)	40	30	40	72	11.3	32	101%
		*p* < 0.001 *	*p* < 0.001 *	*p* < 0.001 *	*p* = 0.606	*p* = 0.109	*p* < 0.001 *	*p* = 0.698
Venous thrombosis		Median	Median	Median	Median	Median	Median	Median
Yes	207 (5.4%)	38	30	46	75	11.9	30.5	94%
No	3605 (94.6%)	41	31	42	72	11.3	32	101%
		*p* = 0.011 *	*p* = 0.608	*p* = 0.665	*p* = 0.035 *	*p* = 0.214	*p* = 0.009 *	*p* < 0.001 *
Arterial thrombosis		Median	Median	Median	Median	Median	Median	Median
Yes	213 (5.6%)	42.5	29	36	74	11.8	32	100%
No	3599 (94.4%)	41	31	42	72	11.4	32	101%
		*p* = 0.688	*p* = 0.204	*p* = 0.061	*p* = 0.461	*p* = 0.664	*p* = 0.225	*p* = 0.848
Bleeding		Median	Median	Median	Median	Median	Median	Median
Yes	304 (8%)	39	29	41	75	11.3	30	98%
No	3508 (92%)	41	31	42	72	11.4	32	101%
		*p* = 0.310	*p* = 0.085	*p* = 0.210	*p* = 0.042 *	*p* = 0.835	*p* < 0.001 *	*p* = 0.018 *
30-day death		Median	Median	Median	Median	Median	Median	Median
Yes	1315 (34.5%)	48	30	43	77.5	12.3	30	97.5%
No	2497 (65.5%)	38	31	41	69	11	33	102%
		*p* < 0.001 *	*p* = 0.812	*p* = 0.199	*p* < 0.001 *	*p* < 0.001 *	*p* < 0.001 *	*p* < 0.001 *

* Statistically significant at level *p* < 0.05; albumin was graded as normal ≥40 g/L, 35–39 g/L and <35 g/L; PT values were considered only in patients not receiving oral anticoagulant therapy.

**Table 3:** Associations of liver blood tests with 30-day survival.

LBT	× ULN	30-Day Survival (%)	Hazard Ratio vs. Normal (95% CI)	*p* for Trend	*p* for Difference
**AST** **(U/L)**	N	74	Reference	<0.001	<0.001
	1–3	63	1.5 (1.33–1.68)		
	>3	50	2.42 (1.97–2.98)		
**ALT** **(U/L)**	N	66	Reference	0.631	0.021
	1–3	68	0.92 (0.82–1.04)		
	>3	59	1.32 (0.99–1.74)		
**GGT** **(U/L)**	N	67	Reference	0.034	0.056
	1–3	66	1.03 (0.92–1.17)		
	>3	61	1.23 (1.03–1.47)		
**ALP** **(U/L)**	N	67	Reference	<0.001	<0.001
	1–3	50	1.72 (1.37–2.14)		
	>3	54	1.61 (0.91–2.85)		
**Bilirubin** **(umol/L)**	N	68	Reference	<0.001	<0.001
	1–3	52	1.73 (1.42–2.09)		
	>3	52	1.79 (1.15–2.78)		
**Albumin** **(g/L)**	>40	89	Reference	<0.001	<0.001
	35–40	80	1.77 (1.29–2.43)		
	<35	57	4.55 (3.39–6.12)		
**PT (%, Quick)**	≥100	73	Reference	<0.001	<0.001
	80–99	66	1.36 (1.19–1.57)		
	<80	56	1.92 (1.52–2.43)		

AST—aspartate aminotransferase, ALT—alanine aminotransferase, GGT—gamma-glutamyl transferase, ALP—alkaline phosphatase, CI—confidence interval, LBT—liver blood tests, PT—prothrombin time (Quick, %), ULN—upper limit of normal.

**Table 4:** Cox regression model investigating independent contribution of investigated parameters to 30-day mortality.

Covariate	*p*	HR	95% CI for HR
Age (years)	<0.001 *	1.0311	1.0207 to 1.0416
Male sex	0.137	1.1593	0.9542 to 1.4086
Obesity	0.796	1.0272	0.8385 to 1.2582
Charlson comorbidity index	<0.001 *	1.1270	1.0831 to 1.1727
COVID severity severe vs. mild	<0.001 *	13.1424	4.1771 to 41.3497
COVID severity critical vs. mild	<0.001 *	28.2629	8.8608 to 90.1489
AST 1–3 × elevated vs. normal	<0.001 *	1.5081	1.2089 to 1.8814
AST >3 × elevated vs. normal	<0.001 *	2.1315	1.3957 to 3.2552
ALT 1–3 × elevated vs. normal	<0.001 *	0.6432	0.5042 to 0.8206
ALT >3 × elevated vs. normal	0.053	0.5762	0.3296 to 1.0072
GGT 1–3 × elevated vs. normal	0.236	1.1393	0.9185 to 1.4131
GGT >3 × elevated vs. normal	0.727	1.0735	0.7207 to 1.5990
ALP 1–3 × elevated vs. normal	0.142	1.3301	0.9088 to 1.9467
ALP >3 × elevated vs. normal	0.129	2.0607	0.8090 to 5.2489
Total bilirubin 1–3 × elevated vs. normal	0.066	1.3087	0.9819 to 1.7443
Total bilirubin >3 × elevated vs. normal	0.881	1.0668	0.4577 to 2.4863
Albumin 35–39 g/L vs. ≥40 g/L	0.759	1.1403	0.4915 to 2.6459
Albumin <35 g/L g/L vs. ≥40 g/L	0.351	1.4787	0.6503 to 3.3624
PT 80–89% vs. ≥100%	0.388	1.0933	0.8929 to 1.3387
PT < 80% vs. ≥100%	0.056	1.3417	0.9920 to 1.8148
Chronic liver disease	0.246	0.5553	0.2057 to 1.4990
Liver cirrhosis	0.610	1.4051	0.3798 to 5.1976

* Statistically significant at level *p* < 0.05. AST—aspartate aminotransferase, ALT—alanine aminotransferase, GGT—gamma-glutamyl transferase, ALP—alkaline phosphatase, CI—confidence interval, PT—prothrombin time (quick, %).

## Data Availability

Data from this investigation are available from the corresponding author upon the reasonable request, after the approval of the University hospital Dubrava who is the founder and the owner of the Registry.

## Supplementary Materials

The following are available online at https://www.mdpi.com/article/10.3390/jcm10184222/s1, Table S1. Patients’ characteristics at admission and their relationship with liver blood tests.
