# Supplementary material for: Prevalence and Prognostic Impact of Deranged Liver Blood Tests in COVID-19: Experience from the Regional COVID-19 Center over the Cohort of 3812 Hospitalized Patients
Source: J Clin Med. 2021 Sep 17;10(18):4222. doi: 10.3390/jcm10184222 (PMC8470265; doi:10.3390/jcm10184222)
Supplement: Supplementary file 1 [file jcm-10-04222-s001.zip › jcm-1348203-supplementary.pdf]

Supplementary Materials

Table S1. Patients' characteristics at admission and their relationship with liver blood tests.

	**Overall /any enzyme**	**AST (U/L)**	**ALT (U/L)**	**GGT (U/L)**	**ALP (U/L)**	**Tot. bilirubin (umol/L)**	**Albumin (g/L)**	**PT (%,Quick)**
Total number of patients	3812	3731	3742	3587	3173	3030	2327	***2235
Normal levels	958 (25.1%)	1432 (38.4%)	2521 (67.4%)	1934 (53.9%)	2862 (90.3%)	2586 (85.3%)	**114 (4.9%)	
1–3 x elevation	2137 (56.1%)	1922 (51.5%)	1049 (28%)	1212 (33.8%)	270 (8.5%)	375 (12.4%)	*515 (22.1%)	2235 (100%)
>3 x elevation	717 (18.8%)	377 (10.1%)	172 (4.6%)	441 (12.3%)	39 (1.2%)	69 (2.3%)	*1698 (73%)	
Age (years)	74 IQR (64–82)	Rho = 0; p = 0.778	Rho = −0.21; p < 0.001 *	Rho = −0.23; p < 0.001 *	Rho = 0.03; p = 0.106	Rho = 0.08; p < 0.001 *	Rho = −0.23; p < 0.001 *	Rho = −0.14; p < 0.001 *
Sex		Median	Median	Median	Median	Median	Median	Median
Female	1664 (43.7%)	37	26	34	73	10.3	31	102%
Male sex	2148 (56.3%)	44	35	49	71	12.2	32	100%
		p < 0.001 *	p < 0.001 *	p < 0.001 *	p = 0.072	p < 0.001 *	p = 0.009 *	p = 0.021 *
Arterial hypertension		Median	Median	Median	Median	Median	Median	Median
Yes	2658 (69.7%)	41	30	41	72	11.4	31	100%
No	1154 (30.3%)	41	32	44.5	71	11.4	32	100%
		p = 0.472	p = 0.008 *	p = 0.010 *	p = 0.764	p = 0.926	p = 0.029 *	p = 0.883
Diabetes mellitus		Median	Median	Median	Median	Median	Median	Median
Yes	1154 (30.3%)	39	29	39	73	11.1	32	100%
No	2658 (69.7%)	42	31	43	72	11.5	32	100%
		p < 0.001 *	p = 0.003 *	p = 0.002 *	p = 0.546	p = 0.049 *	p = 0.693	p = 0.299
Obesity		Median	Median	Median	Median	Median	Median	Median
Yes	1023 (28.6%)	42	34	47	69	11.05	32	103%
No	2554 (71.4%)	40	30	40	73	11.5	31	100%
		p = 0.045 *	p < 0.001 *	p < 0.001 *	p = 0.001 *	p = 0.038 *	p < 0.001 *	p < 0.001 *
Cong. heart failure		Median	Median	Median	Median	Median	Median	Median
Yes	617 (16.2%)	41	25	38	74	12.7	31	92%
No	3195 (82.8%)	41	32	43	71	11.2	32	97%
		p = 0.587	p < 0.001 *	p = 0.006*	p = 0.064	p < 0.001 *	p = 0.007 *	p = 0.013 *
Chr. kidney disease		Median	Median	Median	Median	Median	Median	Median
Yes	474 (12.4%)	38	24	37	77	10.8	30.5	92.5%
No	3338 (87.6%)	41	32	42	71	11.5	32	96%
		p = 0.001 *	p < 0.001 *	p = 0.014 *	p < 0.001 *	p = 0.052	p < 0.001 *	p = 0.114
Chr. liver disease		Median	Median	Median	Median	Median	Median	Median
Yes	106 (2.8%)	57	34	67	91.5	19.1	28	94%
No	3706 (97.2%)	41	31	41	72	11.3	32	101%
		p < 0.001 *	p = 0.402	p < 0.001 *	p < 0.001 *	p < 0.001 *	p < 0.001 *	p = 0.006 *

	Overall /any enzyme	AST (U/L)	ALT (U/L)	GGT (U/L)	ALP (U/L)	Tot. bilirubin (umol/L)	Albumin (g/L)	PT (%,Quick)
Liver cirrhosis<br>Yes<br>No	 49 (1.3%)<br>3763 (98.7%)	Median<br>85<br>41<br>$p < 0.001$ *	Median<br>33<br>31<br>$p = 0.499$	Median<br>84<br>42<br>$p < 0.001$ *	Median<br>103.5<br>72<br>$p < 0.001$ *	Median<br>41.4<br>11.3<br>$p < 0.001$ *	Median<br>25.5<br>32<br>$p < 0.001$ *	Median<br>84%<br>101%<br>$p < 0.001$ *
Charlson comorbidity index	4 IQR (3–6)	Rho = −0.04; $p =$ 0.026 *	Rho = −0.21; $p < 0.001$ *	Rho = −0.13; $p <$ 0.001 *	Rho = 0.12; $p <$ 0.001 *	Rho = 0.056; $p =$ 0.002 *	Rho = −0.27; $p <$ 0.001 *	Rho = −0.17; $p < 0.001$ *
Alcohol use<br>Yes<br>No	 207 (5.4%)<br>3605 (94.6%)	Median<br>46<br>41<br>$p = 0.082$	Median<br>32.5<br>31<br>$p = 0.349$	Median<br>54<br>41<br>$p < 0.001$ *	Median<br>70<br>72<br>$p = 0.674$	Median<br>13.4<br>11.3<br>$p < 0.001$ *	Median<br>31<br>32<br>$p = 0.147$	Median<br>98.5%<br>100%<br>$p = 0.109$
Smoking<br>Yes<br>No	 434 (11.4%)<br>3378 (88.6%)	Median<br>40<br>41<br>$p = 0.550$	Median<br>32.5<br>30<br>$p = 0.015$ *	Median<br>47<br>41<br>$p = 0.008$ *	Median<br>69.5<br>72<br>$p = 0.119$	Median<br>11.6<br>11.3<br>$p = 0.652$	Median<br>32<br>32 ↑<br>$p = 0.019$ *	Median<br>103%<br>101%<br>$p = 0.112$
Number of drugs in chronic therapy	5 IQR (3–8)	Rho = −0.06; $p <$ 0.001 *	Rho = −0.14; $p < 0.001$ *	Rho = −0.07; $p <$ 0.001 *	Rho = 0.08; $p <$ 0.001 *	Rho = −0.02; $p =$ 0.162	Rho = −0.14; $p <$ 0.001 *	Rho = −0.11; $p < 0.001$ *
Statin<br>Yes<br>No	 911 (23.9%)<br>2901 (76.1%)	Median<br>43<br>40<br>$p = 0.112$	Median<br>30<br>31<br>$p = 0.720$	Median<br>37<br>43<br>$p < 0.001$ *	Median<br>69.5<br>73<br>$p = 0.003$ *	Median<br>11.2<br>11.5<br>$p = 0.730$	Median<br>32<br>32<br>$p = 0.180$	Median<br>100%<br>101%<br>$p = 0.378$
Prior antibiotic th.<br>Yes<br>No	 1285 (33.7%)<br>2527 (66.3%)	Median<br>41<br>41<br>$p = 0.480$	Median<br>31.5<br>30<br>$p = 0.083$	Median<br>46<br>40<br>$p < 0.001$ *	Median<br>75<br>70<br>$p < 0.001$ *	Median<br>10.9<br>11.7<br>$p < 0.001$ *	Median<br>30<br>32<br>$p < 0.001$ *	Median<br>99%<br>100%<br>$p = 0.003$ *
Oral anticoagulant th.<br>Yes<br>No	 1049 (27.5%)<br>2763 (72.5%)	Median<br>40<br>41<br>$p = 0.144$	Median<br>27<br>32<br>$p < 0.001$ *	Median<br>43<br>42<br>$p = 0.287$	Median<br>75<br>71<br>$p < 0.001$ *	Median<br>12.2<br>11.1<br>$p < 0.001$ *	Median<br>31<br>32<br>$p < 0.001$ *	-
WBC ($x10^9$/L)	8 IQR (5.8–11.2)	Rho = 0.03; $p <$ 0.037 *	Rho = 0.13; $p < 0.001$ *	Rho = 0.078; $p <$ 0.001 *	Rho = 0.17; $p <$ 0.001 *	Rho = 0.12; $p <$ 0.001 *	Rho = −0.22; $p <$ 0.001 *	Rho = −0.17: $p < 0.001$ *
Hemoglobin (g/L)	128 IQR (113–141)	Rho = 0.16; $p <$ 0.001 *	Rho = 0.22; $p < 0.001$ *	Rho = 0.11; $p <$ 0.001 *	Rho = −0.15; $p < 0.001$ *	Rho = 0.15; $p <$ 0.001 *	Rho = 0.34; $p < 0.001$ *	Rho = 0.22; $p < 0.001$ *
Platelets ($x10^9$/L)	221 IQR (163–297)	Rho = −0.08; $p <$ 0.001 *	Rho = 0.07; $p < 0.001$ *	Rho = 0.06; $p =$ 0.001 *	Rho = 0.12; $p <$ 0.001 *	Rho = −0.14; $p <$ 0.001 *	Rho = −0.08; $p <$ 0.001 *	Rho = −0.02; $p = 0.253$
CRP (mg/L)	88.7 IQR (39.5–151)	Rho = 0.24; $p <$ 0.001 *	Rho = 0.16; $p < 0.001$ *	Rho = 0.17; $p <$ 0.001 *	Rho = 0.08; $p <$ 0.001 *	Rho = 0.06; $p <$ 0.001 *	Rho = −0.34; $p <$ 0.001 *	Rho = −0.11; $p < 0.001$*
Ferritin (ug/L)	711 IQR (386–1289)	Rho = 0.34; $p <$ 0.001 *	Rho = 0.35; $p < 0.001$ *	Rho = 0.31; $p <$ 0.001 *	Rho = 0.03; $p <$ 0.173	Rho = 0.16; $p <$ 0.001 *	Rho = −0.21; $p <$ 0.001*	Rho = −0.01; $p = 0.665$

	Overall /any enzyme	AST (U/L)	ALT (U/L)	GGT (U/L)	ALP (U/L)	Tot. bilirubin (umol/L)	Albumin (g/L)	PT (%,Quick)
D-dimers (mg/L)	1.42 IQR (0.73–3.6)	Rho = 0.06; p = 0.006 *	Rho = −0.01; p = 0.485	Rho = −0.02; p = 0.244	Rho = 0.23; p < 0.001 *	Rho = 0.11; p < 0.001 *	Rho = −0.41; p < 0.001 *	Rho = −0.3; p < 0.001 *
Day of disease on admission	5 IQR (1–9)	Rho = 0.08; p < 0.001 *	Rho = 0.21; p < 0.001 *	Rho = 0.15; p < 0.001 *	Rho = −0.07; p < 0.001 *	Rho = 0; p = 0.987	Rho = −0.05; p = 0.012 *	Rho = 0.08; p < 0.001 *
ECOG status	3 (1–4)	Rho = 0.05; p = 0.001 *	Rho = −0.12; p < 0.001 *	Rho = −0.08; p < 0.001 *	Rho = 0.12; p < 0.001 *	Rho = 0.02; p = 0.148	Rho = −0.33; p < 0.001 *	Rho = −0.17; p < 0.001 *
Pneumonia		Median	Median	Median	Median	Median	Median	Median
Yes	3390 (88.9%)	43	31	43	71	11.4	31	101%
No	422 (11.1%)	29	24	31	78	11.2	35	101%
		p < 0.001*	p < 0.001 *	p < 0.001*	p < 0.001 *	p = 0.296	p < 0.001 *	p = 0.694
Oxygen therapy		Median	Median	Median	Median	Median	Median	Median
Yes	3136 (82.3%)	44	32	44	71	11.5	31	101%
No	676 (17.7%)	31	25	33	78	11.1	35	101%
		p < 0.001 *	p < 0.001 *	p < 0.001 *	p < 0.001 *	p = 0.160	p < 0.001 *	p = 0.956
MEWS severity		Median	Median	Median	Median	Median	Median	Median
Mild	392 (10.3%)	29	24	30	78.5	11.3	35	101%
Moderate	196 (5.1%)	31	26.5	36	76	11.1	34	102%
Severe	2652 (69.6%)	42	31	43	70	11.5	32	102%
Critical	572 (15%)	52	37	52	75	11.4	30	97%
****		p < 0.001 *	p < 0.001 *	p < 0.001 *	p = 0.489	p = 0.246	p < 0.001 *	p = 0.016 *

* statistically significant at level $p < 0.05$; ** albumin was graded as normal ≥40 g/L, 35–39 g/L and <35 g/L; ***PT values were considered only in patients not receiving oral anticoagulant therapy; **** p value for test for trend is presented.
